# Activin receptor-like kinase5 inhibition suppresses mouse melanoma by ubiquitin degradation of Smad4, thereby derepressing eomesodermin in cytotoxic T lymphocytes

**DOI:** 10.1002/emmm.201470040

**Published:** 2014-05-06

**Authors:** Jeong-Hwan Yoon, Su Myung Jung, Seok Hee Park, Mitsuyasu Kato, Tadashi Yamashita, In-Kyu Lee, Katsuko Sudo, Susumu Nakae, Jin Soo Han, Ok-Hee Kim, Byung-Chul Oh, Takayuki Sumida, Masahiko Kuroda, Ji-Hyeon Ju, Kyeong Cheon Jung, Seong Hoe Park, Dae-Kee Kim, Mizuko Mamura



The authors of the above research article have informed the journal that they missed to mention the grant of the Korea Health Technology R&D Project, Ministry of Health & Welfare, Republic of Korea A111345 in the acknowledgements. The new acknowledgements below include this grant.

